# The American Journal of Obstetrics

**Published:** 1871-05

**Authors:** 


The American Journal of Obstetrics.—This journal enters upon its fourth year and fourth volume with the May number, 1871, Of the value of the journal we have repeatedly spoken, and can add nothing new; its character is already fully established. The work is published by William Baldwin & Co., 21 Park Row, New York.

